# Sanitary Matters in Michigan

**Published:** 1888-04

**Authors:** 


### SANITARY MATTERS IN MICHIGAN.

The *State Board of Health* of Michigan is doing good practical sanitary work in that State, which merits commendation. Not the least important of this is the holding of sanitary conventions, under the auspices of that board, in the various cities of the State.

In those conventions are discussed not only the local questions of a sanitary nature, but the subject of sanitary science in general is made familiar, and in a practical way, to citizens who would, but for such opportunities as are afforded by those conventions, give little if any attention to many of the questions that seriously influence the well-being of the community, as well as of the individual. The amount of useful information that is, in this way, conveyed to the masses is great and of incalculable value to them. It not only instructs them in useful methods of guarding against contagious and infectious diseases, but, what is of even greater importance to them, aids them in making intelligent efforts to have their homes and their immediate environment healthful. This plan of diffusing useful knowledge might be profitably imitated by other States.

